# [Corrigendum] Long non‑coding RNA LINC00238 suppresses the malignant phenotype of liver cancer by sponging miR‑522

**DOI:** 10.3892/mmr.2024.13189

**Published:** 2024-02-28

**Authors:** Hong-Gang Qian, Qiong Wu, Jian-Hui Wu, Xiu-Yun Tian, Wei Xu, Chun-Yi Hao

Mol Med Rep 25: 71, 2022; DOI: 10.3892/mmr.2022.12587

Subsequently to the publication of the above article, an interested reader drew to the authors’ attention that two pairs of data panels featured in Figs. 2E and 6D, portraying the results from cell invasion and migration assay experiments, appeared to contain overlapping sections, such that data which were intended to show the results from differently performed experiments had apparently been derived from a smaller number of original sources.

The authors were able to re-examine their original data (which was also presented to the Editorial Office), and realized that errors has been made in the compilation of Fig. 2. The proposed revised version of Fig. 2, now showing the results from the ‘field 1’ view of the data, is shown on the next page. Note that these errors did not significantly affect either the results or the conclusions reported in this paper,. All the authors agree to the publication of this Corrigendum, and are grateful to the Editor of *Molecular Medicine Reports* for allowing them the opportunity to correct this error; furthermore, they apologize to the readership for any inconvenience caused.

## Figures and Tables

**Figure 2.**
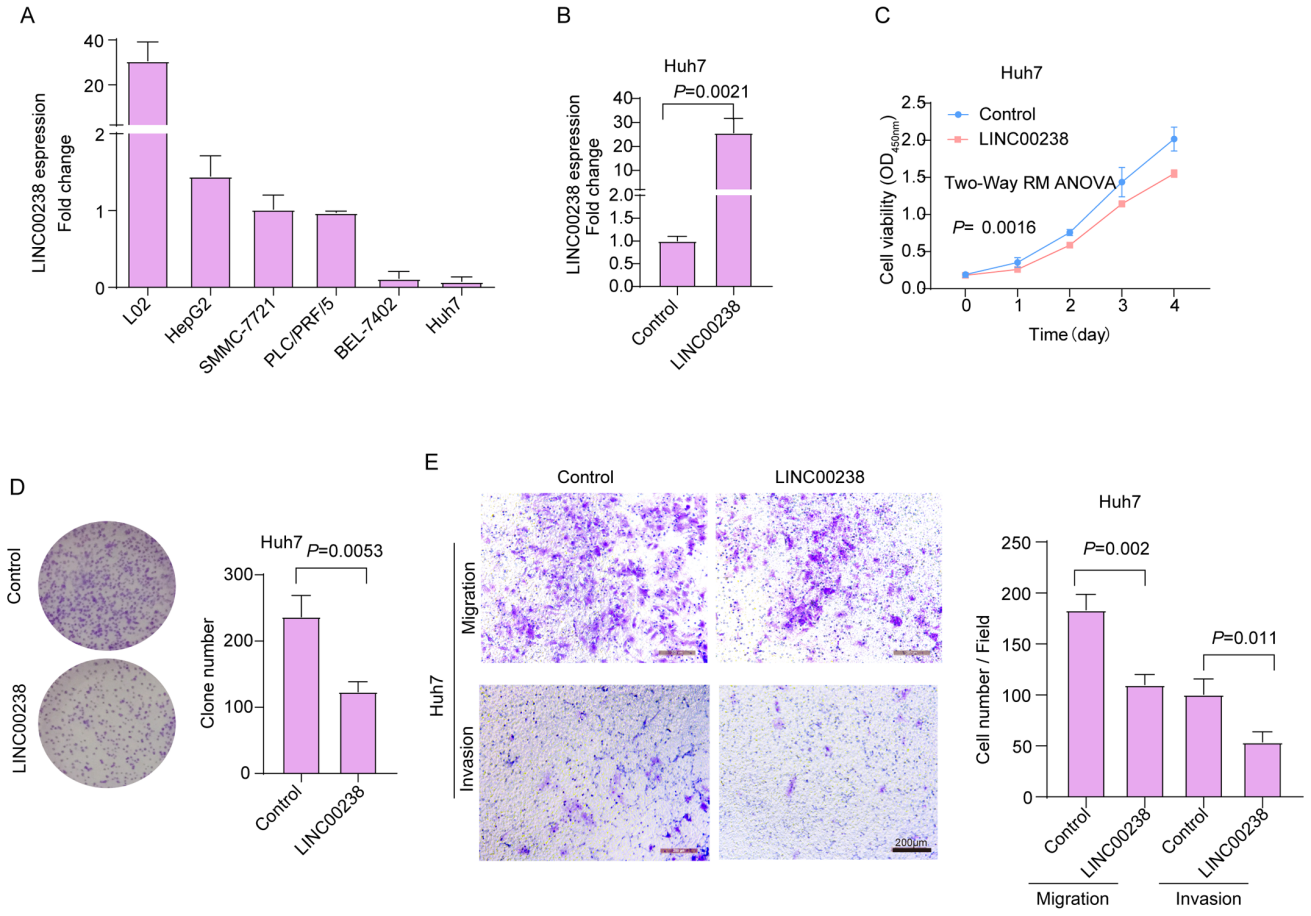
Overexpression of LINC00238 inhibits liver cancer cell viability, invasion and migration *in vitro*. (A,B) Expression levels of LINC00238 in cells were determined via reverse transcription-quantitative PCR. (C) Cell Counting Kit-8 assays indicated that overexpression of LINC00238 suppressed cell viability. (D) Plate colony formation assays showed that overexpression of LINC00238 suppressed cell colony formation. (E) Transwell assays suggested that overexpression of LINC00238 reduced cell migration and invasion. The number of cells was counted in four different fields. Scale bar, 200 µm.

